# Effect of Health Comparisons on Functional Health and Depressive Symptoms - Results of a Population-Based Longitudinal Study of Older Adults in Germany

**DOI:** 10.1371/journal.pone.0156235

**Published:** 2016-05-23

**Authors:** André Hajek, Hans-Helmut König

**Affiliations:** Department of Health Economics and Health Services Research, Hamburg Center for Health Economics, University Medical Center Hamburg-Eppendorf, Hamburg, Germany; University of Vienna, School of Psychology, AUSTRIA

## Abstract

**Objective:**

To investigate the effect of health comparisons on functional health and depressive symptoms in a longitudinal approach. Gender differences were examined.

**Methods:**

The German Ageing Survey (DEAS) is a nationwide, representative longitudinal study of community dwelling individuals living in Germany aged 40 and older. The surveys in 2008 and 2011 were used, with n = 3,983 respondents taking part in both waves. Health comparisons were quantified by the question “How would you rate your health compared with other people your age” (Much better; somewhat better; the same; somewhat worse, much worse). Functional health was assessed by the subscale “physical functioning” of the 36-Item Short Form Health Survey (SF-36) and depressive symptoms were measured by the Center for Epidemiologic Studies Depression Scale (CES-D).

**Results:**

Adjusting for sociodemographic factors, self-assessed health, social network, self-efficacy and optimism, and morbidity, fixed effects regressions revealed that functional health decreased significantly and considerably with negative health comparisons in the total sample (transitions from ‘the same’ to ‘much worse’: β = -11.8), predominantly in men. The effects of negative health comparisons (transitions from ‘the same’ to ‘much worse’: β = 4.8) on depressive symptoms were comparable (in terms of significance) to the effects on functional health, with stronger effects in women. Positive comparisons did not affect functional health and depressive symptoms.

**Conclusion:**

Our findings underline the relevance of negative health comparisons on functional health (men) and depressive symptoms (women). Comparison effects are asymmetric and mostly upwards.

## Introduction

According to the Easterlin-Paradox [1–3] richer individuals are more satisfied than poorer individuals in the same country at a given point in time. However, income increases (or increases in economic growth) do not lead to increases in subjective well-being in industrialized countries in the long run. A possible interpretation of this seeming paradox might be that the well-being does not only depend on the absolute income, but additionally on the income in relative terms. The perception of the individual income might be affected by the individual’s own income in the past and the income compared with other individuals (reference group, e. g. colleagues or subjects with the same academic background). The latter one is often referred as “comparison income” or “relative utility”. In most cases, it is assumed that the individuals are more satisfied the larger their income is in comparison with the income of a reference group in industrial countries. Additionally, it is supposed that while the well-being of individuals is negatively affected by an income below that of their reference group, individuals with an income above that of their reference group do not gain satisfaction (asymmetric effects [4,5])—an idea which was introduced by Duesenberry in 1949 [4]. In other words, income comparisons are supposed to be mostly upwards. So far, only few empirical studies investigated the effect of individual income comparisons and asymmetric states on well-being [2,4–14]. According to these studies, economic comparisons are mostly upwards and differ by gender, with men being more strongly affected by economic upwards comparisons.

The existing studies mainly focused on the effect of income comparisons on subjective well-being. Furthermore, other studies found that chronically ill patients often make comparisons with each other [15]. These comparisons might help to reduce anxiety (for example, by making downward comparisons [16]). Additionally, comparisons might affect physical health [15]. For example, upwards comparisons (if others are better off) might lead to negative emotions such as frustration [15,17] or anger which in turn can affect functional health [18] or health-related quality of life [19]. Moreover, comparisons might affect motivation for self-care [15] which in turn might affect health behavior [20]. In conclusion, we hypothesize that the idea of income comparisons can be extended to the domain of health.

In medical research, there is a stronger focus on functional health and depressive symptoms since both are associated with numerous negative health consequences, such as mortality [16] or the risk of institutionalization [21,22]. One of the major predictors of both, functional and depressive symptoms, is self-rated health [23]. Quite analogously to individual income, it is assumed that individual self-rated health is subject to the past own situation and individual’s own health compared to the health of other individuals (e.g. individuals in the same age bracket), referring to it as ‘health comparisons’.

In this context, to our knowledge no study has investigated the effect of health comparisons on functional health and depressive symptoms thus far. Therefore, we aimed at investigating the effect of health comparisons on (1a) functional health as well as (1b) depressive symptoms, drawing on a representative sample of individuals aged 40 and older in a longitudinal setting. Moreover, we aimed at determining whether (2) negative and positive health comparisons affect the outcome variables differently (asymmetric effects). Additionally, (3) gender-specific effects were estimated.

In total, our hypotheses are as follows: (1) Health comparisons affect (i) functional health as well as (ii) depressive symptoms. (2) Negative health comparisons affect the outcome variables more strongly than positive health comparisons. (3) The effect of health comparisons on the outcome variables is more pronounced in men.

Fixed effects (FE) regressions were used to estimate the effect of health comparisons on functional health and depressive symptoms, adjusting for numerous regressors often found to be relevant for our outcome variables, such as sociodemographic factors [24,25], social network, self-efficacy and optimism [26], as well as morbidity [27]. By using panel-econometric techniques, unobserved heterogeneity (e. g. personality or genetic disposition) can be taken into account.

## Methods

### Sample

Data were derived from the third and fourth wave from the public release of the German Ageing Survey (DEAS), provided by the Research Data Centre of the German Centre of Gerontology (DZA). It is a population-based, representative survey of the German population aged 40 and older where individuals were interviewed in their homes by trained staff using a standardized questionnaire. The sampling procedure reflects a national probability sampling. While 8,200 individuals participated in the third wave, 4,855 individuals participated in the fourth wave, thereof 3,983 respondents took part in both waves and filled out the functional health and depressive symptoms questions. These 3,983 individuals were included in our analysis.

Discrepancies regarding sample sizes can mainly be explained by the collection of new samples in the third wave. Thereby, 6,205 community-dwelling individuals (birth cohorts 1923–1968) participated for the first time in the third wave, whereas 1,995 individuals had already been interviewed in the former waves. The fourth wave is a “pure” panel survey. Most of the individuals included in the fourth wave belong to the panel sample 2008. More details regarding the sampling frame and the sample composition have been reported by Engstler and Motel-Klingebiel [28]. Written informed consent was given prior to the interview.

#### Functional health and depressive symptoms

*Functional health* was assessed by the subscale “physical functioning” of the 36-Item Short Form Health Survey (SF-36) [29]. Impairments in ten activities of daily living such as climbing stairs, bathing or lifting or carrying groceries were rated on a three-point scale [30]. Consequently, we focus on rather simple activities of daily living. The items were transformed into a scale ranging from 0 (worst score) to 100 (best score).

*Depressive symptoms* was quantified by the Center for Epidemiologic Studies Depression Scale (CES-D) [31], consisting of 15 items. The scale represents the sum of all 15 items (0–45), with high values indicating more depressive symptoms.

#### Health comparison

*Health comparisons* were quantified by the question “How would you rate your health compared with other people your age” (Much better; somewhat better; the same; somewhat worse; much worse). The category 'the same' was considered as reference category.

#### Potential confounders

Furthermore, we controlled for time-dependent regressors which might be relevant for functional health and depressive symptoms. Thus, we controlled for *age*, *family status* (Ref.: married, living together with spouse; married, living separated from spouse; divorced; widowed; never married), *monthly household net income* in Euro (logarithmized) and *morbidity* (total number of physical diseases, ranging from 0 to 10, e. g. cardiovascular diseases, diabetes, cancer, respiratory diseases, eye diseases, hearing problems). These conditions were informed by the Charlson Comorbidity Index [32]. Moreover, we controlled for the *number of important people in regular contact* (0–9) and *self-efficacy and optimism* (HOPE scale [33]), ranging from 1–4 (high values indicate great self-efficacy) since it has been shown that self-efficacy is a predictor of our outcome measures [34,35]. Additionally, we included dummy coded variables for *region* and *employment status* (Ref.: working; retired; other: not employed) (both not shown, but available upon request).

Furthermore, for descriptive purposes, the time-invariant sociodemographic variable *education* (level of education by ISCED-97 (International Standard Classification of Education [36]) with three categories: low (0–2), medium (3–4) and high (5–6)) was used.

In order to rule out the possibility that our health comparisons only reflect deteriorations in own subjective health, we additionally controlled for *subjective health* (1 = “very good” to 5 = “very bad”).

### Statistical analyses

Compared with cross sectional regression techniques, longitudinal regression techniques offer the advantage of controlling for unobserved heterogeneity, such as genetic disposition. This is important in well-being research as unobserved heterogeneity is in most cases systematically correlated with the regressors. In such a case, random effects (RE) regressions are inconsistent. Thus, FE regressions which leads to consistent estimates are the method of choice since unobserved heterogeneity can be taken into account [37]. It is worth mentioning that in FE regressions, solely within-variations are used (hence, also called ‘within-estimator’). Thus, only time-dependent variables can be included in FE regression models. Standard errors that cluster errors at the individual level were reported in order to account for heteroscedasticity and serial correlation [38].

A panel regression model can be denoted as follows [39]:

$$Y_{it}=\alpha_{i}+\beta X_{it}+\gamma_{i}W_{i}+\lambda_{t}+\varepsilon_{it}$$

i = 1, …, N: units (persons); t = 1,…, T: time

Factors changing over time (but constant across individuals) are denoted as λ_t_. Constant observed characteristics of individuals are denoted as W_i_. Time-varying idiosyncratic errors are denoted as ε_it_ and time-dependent predictors are denoted as X_it_. Contrary to cross-sectional regressions, an individual specific intercept α_i_ is included in this model. This factor captures the impact of unobserved factors constant over time of an individual I on outcomes Y_it_. This is especially important when a correlation between observed predictors and α_i_ is allowed (- addressing the endogenous selection into treatment—based on time-constant unobserved factors). This is achieved by the FE-estimator.

The FE-estimator used within-transformed data to estimate the equation mentioned above from variation in observed independent variables and outcome variables (intraindividual changes over time):

$$Y_{it}-{\bar{Y}}_{i}=\beta \left(X_{it}-{\bar{X}}_{i}\right)+\lambda_{t}-\bar{\lambda }+\left(\varepsilon_{it}-{\bar{\varepsilon }}_{i}\right)$$

By differencing the data, the impact of time-constant factors (both unobserved and observed) is removed. Hence, changes in the dependent variable (Y_it_−${\bar{Y}}_{i}$) merely depend on changes in time-varying predictors X_it_ as well as time-varying idiosyncratic errors ε_it_.

Furthermore, it is worth mentioning that the Stata command for FE regression analysis include individuals with only one observation in calculating the number of observation as they provide information about the constant and the variance components and so forth. Nevertheless, it does not affect the standard errors and the beta-coefficients.

## Results

### Descriptive analysis

For individuals included in FE regressions, our variables are described over time (waves 3–4) in Table 1. As for FE regressions with functional health as outcome variable, at wave 3, mean age was 62.1 years (±10.9 years), ranging from 40 to 93 years. The majority was male (52.3%), was married, living together with spouse (74.7%), had medium education (51.1%), and was retired (50.7%). The mean monthly household net income was €2,703.7 (±€2,978.9). The mean number of important people in regular contact was 4.9 (±2.8) and mean HOPE scale was 3.1 (±0.4). Moreover, the mean number of physical diseases was 2.3 (±1.8) and mean self-assessed health was 2.4 (±0.8). As for the key independent variable, health comparisons, the majority rated their health as ‘the same’ (31.3%) or ‘somewhat better’ (41.1%) compared with other people their age. Mean functional health score was 86.3 (±19.2). After 3 years (wave 4), the proportion of retired individuals increased to 58.3%. Other variables remained nearly constant. Descriptive statistics for individuals included in FE regressions with depressive symptoms as outcome variable were almost the same. Besides, mean depressive symptoms score was 6.0 (±5.8) at wave 3.

**Table 1:** Descriptive statistics over time (for individuals included in FE regressions; with functional health as well as depressive symptoms as outcome variable, Waves 3–4).

	Functional health	Functional health	Depressive symptoms	Depressive symptoms
	Wave 3 (n = 2,240)	Wave 4 (n = 2,240)	Wave 3 (n = 2,201)	Wave 4 (n = 2,201)
Age: Mean (SD)	62.1 (10.9)	65.1 (10.9)	62.0 (10.9)	65.0 (10.9)
Female: N (%)	1,067 (47.7)	1,067 (47.7)	1,049 (47.7)	1,049 (47.7)
Marital status: N (%)				
Married, living together with spouse	1,673 (74.7)	1,633 (72.9)	1,645 (74.7)	1,610 (73.1)
Married, living separated from spouse	25 (1.1)	28 (1.2)	25 (1.1)	28 (1.3)
Divorced	194 (8.7)	195 (8.7)	192 (8.7)	192 (8.7)
Widowed	226 (10.1)	266 (11.9)	219 (10.0)	255 (11.6)
Single	122 (5.4)	118 (5.3)	120 (5.5)	116 (5.3)
Level of education (ISCED categories): N (%)				
Low (0–2)	158 (7.1)	158 (7.1)	152 (6.9)	152 (6.9)
Medium (3–4)	1,144 (51.1)	1,144 (51.1)	1,131 (51.4)	1,131 (51.4)
High (5–6)	937 (41.8)	937 (41.8)	917 (41.7)	917 (41.7)
Employment status: N (%)				
Working	835 (37.3)	728 (32.5)	829 (37.7)	720 (32.7)
Retired	1,135 (50.7)	1,306 (58.3)	1,108 (50.3)	1,276 (58.0)
Other: not employed	270 (12.0)	206 (9.2)	264 (12.0)	205 (9.3)
Monthly household net income in Euro: Mean (SD)	2,703.7 (2,978.9)	2,717.1 (2,095.2)	2,706.3 (2,999.4)	2720.1 (2,104.8)
Number of important people in regular contact: Mean (SD)	4.9 (2.8)	5.1 (2.7)	4.9 (2.8)	5.1 (2.7)
Self-efficacy and optimism (HOPE Scale): Mean (SD)	3.1 (0.4)	3.0 (0.4)	3.1 (0.4)	3.1 (0.4)
Morbidity (total number of physical diseases): Mean (SD)	2.3 (1.8)	2.5 (1.8)	2.3 (1.7)	2.5 (1.8)
Self-assessed health: Mean (SD)	2.4 (0.8)	2.5 (0.8)	2.4 (0.8)	2.5 (0.8)
Health comparison				
Much worse	45 (2.0)	51 (2.3)	44 (2.0)	49 (2.2)
Somewhat worse	211 (9.4)	190 (8.5)	209 (9.5)	185 (8.4)
The same	701 (31.3)	717 (32.0)	690 (31.4)	707 (32.1)
Somewhat better	920 (41.1)	930 (41.5)	907 (41.2)	915 (41.6)
Much better	363 (16.2)	352 (15.7)	351 (15.9)	345 (15.7)
Functional health (Subscale 'Physical Functioning' of the SF-36): Mean (SD)	86.3 (19.2)	82.9 (22.3)		
Depressive symptoms (CES-D): Mean (SD)			6.0 (5.8)	6.4 (5.9)

SD: Standard deviation

In men, the pooled mean depressive symptoms score was 5.5 (±5.1) and the pooled functional health score was 86.2 (±19.7), whereas in women, the pooled mean depressive symptoms score was 6.9 (±6.5) and the pooled functional health score was 82.9 (±22.0).

### Regression analysis

Findings of the longitudinal regressions are depicted in Table 2 (functional health) and Table 3 (depressive symptoms). Since we were interested in the intraindividual changes, individuals were only included in the sample if they had changes in the outcome variable between the third and fourth wave. Consequently, 4,184 participants were interviewed in the third and fourth wave. Thereof, 2,240 individuals were included in FE regressions with functional health as outcome variable. Moreover, 2,201 individuals were included in FE regressions with depressive symptoms as outcome variable. The slight differences between the different regressions can be explained by missing values in depressive symptoms.

**Table 2:** Longitudinal predictors of functional health (Subscale 'Physical Functioning' of the SF-36): Results of fixed effects regressions (Waves 3–4) in (1) total sample, (2) men and (3) women.

	(1)	(2)	(3)
Independent variables	Functional health—Total sample	Functional health—Men	Functional health—Women
Age	-0.978***	-1.075***	-0.880***
	(0.113)	(0.146)	(0.177)
Married, living separated from spouse (Ref.: married, living together with spouse)	-0.446	-3.825*	3.677
	(3.000)	(1.591)	(6.732)
Divorced	-2.614	-1.069	-3.388
	(2.886)	(3.374)	(5.099)
Widowed	-1.082	-3.316	-0.526
	(2.285)	(3.471)	(3.069)
Never married	-1.875	-0.636	-3.497
	(2.902)	(3.423)	(6.218)
Monthly household net income in Euro (logarithmized)	-1.481	0.440	-2.871
	(1.595)	(1.367)	(2.852)
Number of important people in regular contact	-0.0929	-0.120	-0.0465
	(0.103)	(0.127)	(0.167)
Self-efficacy and optimism (HOPE Scale)	3.731***	4.022**	3.467*
	(1.024)	(1.347)	(1.520)
Morbidity (total number of physical diseases)	-0.183	-0.552	0.156
	(0.254)	(0.370)	(0.339)
Self-assessed health	-4.766***	-4.584***	-4.584***
	(0.550)	(0.755)	(0.755)
Health comparison: Somewhat worse (Ref.: The same)	-2.374+	-3.424*	-1.348
	(1.217)	(1.732)	(1.710)
Health comparison: Much worse	-11.80***	-12.92**	-9.972*
	(3.138)	(4.699)	(4.250)
Health comparison: Somewhat better	0.878	-0.113	1.734+
	(0.661)	(0.966)	(0.885)
Health comparison: Much better	1.242	0.374	1.993
	(0.931)	(1.314)	(1.315)
Constant	153.2***	135.3***	145.0***
	(12.15)	(13.39)	(22.08)
Observations	4,480	2,344	2,134
Number of Individuals	2,240	1,172	1,067
R^2^	0.134	0.161	0.120

Comments: Beta-Coefficients were reported; Cluster-robust standard errors in parentheses; Regressions are also controlled for region and employment status;

*** p<0.001,

** p<0.01,

* p<0.05,

^+^ p<0.10;

Observations with missing values were dropped (listwise deletion).

**Table 3:** Longitudinal predictors of depressive symptoms (CES-D): Results of fixed effects regressions (Waves 3–4) in (1) total sample, (2) men and (3) women.

	(1)	(2)	(3)
Independent variables	Depressive symptoms—Total sample	Depressive symptoms—Men	Depressive symptoms—Women
Age	0.128**	0.132*	0.125+
	(0.0435)	(0.0524)	(0.0711)
Married, living separated from spouse (Ref.: married, living together with spouse)	-2.113	-0.0430	-4.832
	(2.192)	(1.959)	(4.375)
Divorced	2.040	-0.398	3.891
	(2.162)	(1.669)	(4.186)
Widowed	0.758	-0.595	1.862
	(1.315)	(1.692)	(1.753)
Never married	-0.334	-0.989	1.708
	(1.762)	(1.652)	(4.591)
Monthly household net income in Euro (logarithmized)	-0.297	-0.751	-0.0625
	(0.464)	(0.606)	(0.666)
Number of important people in regular contact	-0.0622	-0.0112	-0.120+
	(0.0391)	(0.0464)	(0.0654)
Self-efficacy and optimism (HOPE Scale)	-1.489***	-1.959***	-1.078+
	(0.408)	(0.563)	(0.566)
Morbidity (total number of physical diseases)	0.139	0.184	0.120
	(0.0932)	(0.121)	(0.142)
Self-assessed health	1.759***	1.666***	1.791***
	(0.209)	(0.257)	(0.332)
Health comparison: Somewhat worse (Ref.: The same)	0.847+	0.500	1.325
	(0.492)	(0.529)	(0.817)
Health comparison: Much worse	4.767***	3.973*	5.431**
	(1.203)	(1.731)	(1.692)
Health comparison: Somewhat better	-0.238	-0.294	-0.229
	(0.240)	(0.306)	(0.368)
Health comparison: Much better	-0.265	0.361	-0.953+
	(0.361)	(0.452)	(0.559)
Constant	-6.015	2.045	-3.294
	(4.810)	(6.093)	(6.401)
Observations	4,402	2,302	2,098
Number of Individuals	2,201	1,151	1,049
R²	0.106	0.116	0.118

Comments: Beta-Coefficients were reported; Cluster-robust standard errors in parentheses; Regressions are also controlled for region and employment status;

*** p<0.001,

** p<0.01,

* p<0.05,

^+^ p<0.10;

Observations with missing values were dropped (listwise deletion).

In the total sample, the occurrence of negative health comparisons was significantly associated with substantial losses in functional health (transitions to ‘much worse’: β = -11.8), while the occurrence of positive health comparisons was not significantly associated with improvements in functional health. Gender-specific analysis showed that negative health comparisons were associated with considerable losses in functional health in men and in women (men: transitions to ‘somewhat worse’: β = -3.4; transitions to ‘much worse’: β = -12.9; women: transitions to ‘much worse’: β = -10.0).

Furthermore, in the total sample and in both sexes, functional health decreased with age and decreases in self-assessed health. Moreover, functional health decreased with less self-efficacy (HOPE scale) in the total sample and in both sexes. As for marital status, only the transition from ‘married, living together with spouse’ to ‘married, living separated from spouse’ affected functional health in men significantly. Other potential confounders did not achieve statistical significance.

In the total sample and in both sexes, the occurrence of negative health comparisons was significantly associated with more depressive symptoms (transitions to ‘much worse': β = 4.8; men: β = 4.0; women: β = 5.4), while the occurrence of positive health comparisons was not significantly associated with less depressive symptoms.

Furthermore, in the total sample, depressive symptoms increased with a decrease in self-assessed health. In the total sample and in men, depressive symptoms increased with age and less self-efficacy (HOPE scale). In total sample and in both sexes, all other potential confounders did not affect depressive symptoms significantly.

### Additional analysis

We also estimated the effect of transitions from 'the same or better (= somewhat or much better)' (0) to 'somewhat worse' (1) or 'much worse' (2). Moreover, we estimated the effect of transitions from 'the same or worse' (= somewhat or much worse) (0) to 'somewhat better' (1) or 'much better' (2). In terms of effect size and significance, findings were mostly the same. However, in this model positive comparisons slightly increased functional health in women (transitions to ‘somewhat better’: β = 1.9) and the number of important people in regular contact was significantly associated with depressive symptoms in women (results of additional analysis are not shown, but are available upon request).

## Discussion

### Main findings

Longitudinal regressions revealed that functional health decreased significantly and considerably with negative health comparisons in the total sample (transitions from ‘the same’ to ‘much worse’: β = -11.8), predominantly in men.

In total sample and in both sexes, the occurrence of negative health comparisons was significantly associated with increased depressive symptoms (transitions from ‘the same’ to ‘much worse': β = 4.8), with stronger effects in women. The occurrence of positive health comparisons was not significantly associated with improvements in both outcome measures.

### Previous research

It is difficult to compare our study with previous studies, since this is—to our knowledge—the first study examining the effect of health comparisons on functional health and depressive symptoms. Nevertheless, our finding that individuals weight upward comparisons more heavily than downward comparisons is filled with evidence from cross-sectional [14,40] and longitudinal studies [7,9] using micro data sets. It is worth repeating that these studies referred to *income* comparisons and measures of happiness.

The slight gender differences in our study might be explained by the fact that women might more heavily rely on mental rather than functional aspects by taking health comparisons, whereas men might heavily rely on functional aspects in the process of making health comparisons in mid and late life. This is supported by a study detecting associations between self-assessed health and depressive symptoms in old age women in Germany [41]. Moreover, evidence is present concerning the predictive value of global health on functional decline in old men [42], mostly underlining our findings.

### Strengths and limitations

To our knowledge, this is the first study analyzing the effect of health comparisons on functional health as well as depressive symptoms in Germany. Moreover, asymmetries between upwards and downwards health comparisons were examined.

Additionally, by using longitudinal data, insights into the mechanisms can be derived and unobserved heterogeneity can be taken into account by using FE regressions. Furthermore, a key strength is that we draw on a representative sample of individuals aged 40 and older living in Germany.

One limitation might be that the corresponding reference group for health comparisons is explicitly listed (age-bracket). Therefore, we cannot rule out the possibility that other reference groups (e. g. colleagues or individuals having the same sex)–as indicated in income comparisons [43,44]–might be more relevant for health comparisons or at least might also enter the comparison process [45]. However, we strongly assume that the age bracket is the most salient dimension for health comparisons. Moreover, even though the face validity of this item seems to be acceptable, future research is required to ensure its validity. Another limitation is that our estimates might be slightly biased downwards due to panel mortality (endogeneity selection bias) in the German Ageing Survey between the third and fourth wave [46]. For example, the probability of participation in the fourth wave is affected by sex, education or self-assessed health.

## Conclusion

Our findings underline the relevance of negative health comparisons for functional health (men) and depressive symptoms (women). Comparison effects are asymmetric and primarily upwards. Furthermore, the concept of comparisons might be more general and can be extended to the area of health.

More research is required in this area. For instance, personality [47,48] might moderate the relationship of comparisons and functional health as well as depressive symptoms. Even adaptation might play a role in this area [49–51].
